# Cost-effectiveness of Xpert MTB/RIF for tuberculosis diagnosis in South Africa: a real-world cost analysis and economic evaluation

**DOI:** 10.1016/S2214-109X(17)30205-X

**Published:** 2017-06-12

**Authors:** Anna Vassall, Mariana Siapka, Nicola Foster, Lucy Cunnama, Lebogang Ramma, Katherine Fielding, Kerrigan McCarthy, Gavin Churchyard, Alison Grant, Edina Sinanovic

**Affiliations:** a TB Centre, London School of Hygiene & Tropical Medicine, London, UK; b Department of Global Health and Development, London School of Hygiene & Tropical Medicine, London, UK; c Health Economics Unit, School of Public Health and Family Medicine, University of Cape Town, Cape Town, South Africa; d Aurum Institute, Johannesburg, South Africa; e School of Public Health, Faculty of Health Sciences, University of the Witwatersrand, Johannesburg, South Africa; f Division of Public Health, Surveillance and Response, National Institute for Communicable Disease of the National Health Laboratory Service, Johannesburg, South Africa; g Advancing Treatment and Care for TB/HIV, South African Medical Research Council, Johannesburg, South Africa; h Africa Health Research Institute, School of Nursing and Public Health, University of KwaZulu-Natal, Durban, South Africa

## Abstract

**Background:**

In 2010 a new diagnostic test for tuberculosis, Xpert MTB/RIF, received a conditional programmatic recommendation from WHO. Several model-based economic evaluations predicted that Xpert would be cost-effective across sub-Saharan Africa. We investigated the cost-effectiveness of Xpert in the real world during national roll-out in South Africa.

**Methods:**

For this real-world cost analysis and economic evaluation, we applied extensive primary cost and patient event data from the XTEND study, a pragmatic trial examining Xpert introduction for people investigated for tuberculosis in 40 primary health facilities (20 clusters) in South Africa enrolled between June 8, and Nov 16, 2012, to estimate the costs and cost per disability-adjusted life-year averted of introducing Xpert as the initial diagnostic test for tuberculosis, compared with sputum smear microscopy (the standard of care).

**Findings:**

The mean total cost per study participant for tuberculosis investigation and treatment was US$312·58 (95% CI 252·46–372·70) in the Xpert group and $298·58 (246·35–350·82) in the microscopy group. The mean health service (provider) cost per study participant was $168·79 (149·16–188·42) for the Xpert group and $160·46 (143·24–177·68) for the microscopy group of the study. Considering uncertainty in both cost and effect using a wide range of willingness to pay thresholds, we found less than 3% probability that Xpert introduction improved the cost-effectiveness of tuberculosis diagnostics.

**Interpretation:**

After analysing extensive primary data collection during roll-out, we found that Xpert introduction in South Africa was cost-neutral, but found no evidence that Xpert improved the cost-effectiveness of tuberculosis diagnosis. Our study highlights the importance of considering implementation constraints, when predicting and evaluating the cost-effectiveness of new tuberculosis diagnostics in South Africa.

**Funding:**

Bill & Melinda Gates Foundation.

## Introduction

In 2014, 1·5 million people died from tuberculosis, of whom 25% had HIV and 13% had multidrug-resistant tuberculosis.^1^ Correctly identifying tuberculosis in a timely manner remains a central programmatic challenge. The diagnosis of tuberculosis in most settings with a high tuberculosis burden is based on sputum smear microscopy. Sputum smear microscopy has a limited sensitivity, especially in people living with HIV/AIDS, and cannot distinguish multidrug-resistant tuberculosis.^2^ This poor performance, together with inadequate access to laboratory facilities in many low-income and middle-income countries, contributes to low levels of tuberculosis case detection globally, particularly in countries with a high prevalence of HIV. In 2010, a new test, Xpert MTB/RIF (Cepheid, Sunnyvale, CA, USA), received a guideline recommendation from WHO for use as an initial diagnostic test for tuberculosis.^3^ A Cochrane systematic review^4^ found that Xpert has a specificity of 98% and a sensitivity of 88%, and has 80% sensitivity for people living with HIV, compared with the gold standard of culture. Xpert also provides a signal for rifampicin-resistant tuberculosis, a marker for multidrug-resistant tuberculosis. Since the development of Xpert, several other new tuberculosis diagnostic technologies have emerged or are anticipated in the coming years.

Many model-based economic evaluations,^5^, ^6^, ^7^ predicted that Xpert would be cost-effective, either through a reduction in tuberculosis-related mortality or reduction in the overtreatment of tuberculosis, or both, in a wide range of settings. Nevertheless, concerns over cost-effectiveness remained and the WHO guideline recommendation was conditional, explicitly acknowledging potential resource implications. An individually randomised clinical trial (TB-NEAT)^8^ suggested that high levels of tuberculosis treatment without bacteriological confirmation (ie, empirical treatment) might result in Xpert having a limited effect on tuberculosis-related mortality in practice in South Africa.^9^ Applying these results to their original model, Menzies and colleagues^9^ found Xpert would still be cost-effective, partly due to a reduction in treatment costs, although cost-effectiveness was reduced from earlier estimates.

**Research in context**

**Evidence before this study**

We searched PubMed for studies published in English between Jan 1, 2010, and June 30, 2016, using the terms “tuberculosis” AND “cost” AND (“Xpert*” OR “Genexpert” OR “MTB/RIF”). Several previous studies have been done to estimate the cost-effectiveness of Xpert MTB/RIF using models. These studies all found Xpert MTB/RIF to be cost-effective, with the most recent study estimating incremental cost per disability-adjusted life-year (DALY) averted at US$1208 (95% posterior intervals 621–3995) in South Africa. No previous studies were found that assessed the cost-effectiveness of Xpert MTB/RIF (in terms of cost per DALY averted) using primary data on costs and outcomes in routine settings.

**Added value of this study**

This study provides robust evidence that suggests Xpert MTB/RIF did not increase the costs or improve the cost-effectiveness of tuberculosis investigation, diagnosis, and treatment in the context of routine implementation in South Africa, for a cohort of people being investigated for tuberculosis. The study provides an illustration of the potential effect of real-world implementation on estimates of the cost-effectiveness of new diagnostics.

**Implications of all the available evidence**

While new tuberculosis diagnostics continue to offer substantial potential, this study, along with other research from South Africa and Brazil, suggests that the incremental costs, effect, and cost-effectiveness of Xpert MTB/RIF might be fundamentally affected by real-world issues such as empirical treatment practices, availability of HIV treatment, and provider and patient adherence. We recommend that future investments in tuberculosis diagnostics (and the analysis used to inform them) should explicitly consider and reflect uncertainty (and the additional costs) of implementation constraints, the tuberculosis cascade, and the availability of complementary interventions.

In 2012, the decision was made to rapidly roll out Xpert in South Africa at scale.^10^ The National Department of Health, South Africa agreed to stage the roll-out of Xpert to allow for a pragmatic cluster-randomised trial, the XTEND study,^11^ to assess the effect on mortality and cost-effectiveness of Xpert's introduction across South Africa. The XTEND study found no evidence that the Xpert introduction reduced 6 month mortality risk in adult clinic attendees being investigated for tuberculosis^11^ as Xpert was scaled up nationally. We present a cost analysis and economic evaluation comparing Xpert with sputum smear microscopy as an initial diagnosis for tuberculosis. We analysed an extensive set of primary data, which was collected as Xpert was rolled out in South Africa, to estimate real-world costs and cost-effectiveness. In doing so, we aimed to improve the existing economic analysis used to inform investment in tuberculosis diagnostics.

## Methods

### Study design and participants

For this real-world cost analysis and economic evaluation, we analysed data collected from 40 primary health facilities during the XTEND study,^11^ a pragmatic cluster-randomised trial, in which 20 clusters (defined as laboratories) in four provinces in South Africa (Gauteng, Mpumalanga, Free State, and Eastern Cape) were randomly assigned into Xpert or sputum smear microscopy groups, stratified by province. At two primary care clinics per laboratory, a systematic sample of adults aged 18 years or older and systematically selected by health service staff as needing to provide sputum for tuberculosis investigation were enrolled. 4972 people were screened for study inclusion, and 4656 (94%) were enrolled and followed up for 6 months (median age 36 years; 2891 [62%] of 4656 were female; 2206 [62%] of 3542 who were tested and willing to share results reported being HIV-positive). The Xpert group were less likely to have a body-mass index of less than 18·5 kg/m^2^ and to report tuberculosis symptoms.^11^ Further details of the study population can be found in the appendix.

Participants of the XTEND study were enrolled between June 8, and Nov 16, 2012, and follow-up ended on May 17, 2013. The timeframe for our economic analysis and cost data collection was within trial from enrolment until the 6 month follow-up interview. This timeframe excludes three factors that potentially could affect cost-effectiveness after the 6 month follow-up. First, by reducing time with tuberculosis, Xpert might reduce the transmission of tuberculosis. However, the XTEND study^11^ found no evidence for differences in time to treatment, the factor most likely to affect transmission. Second, increased diagnosis and survival from tuberculosis might increase antiretroviral therapy costs. However, no increase in the number of people initiating antiretroviral therapy was reported during XTEND. Third, and most importantly, XTEND might improve outcomes by hastening the time to effective treatment in people with multidrug-resistant tuberculosis. XTEND was not powered to examine differences in time to multidrug-resistant tuberculosis treatment initiations; as such, the study provides no new evidence on the effect of Xpert on the treatment and outcomes of people with multidrug-resistant tuberculosis. However, intuitively, the identification of rifampicin resistance might improve the time to treatment of people with multidrug-resistant tuberculosis; consequently, previous economic evaluations use models to investigate the potential for Xpert to improve outcomes over a period longer than 6 months.

We examined the emerging evidence from the Xpert roll-out in South Africa to ascertain whether the inclusion of these potential outcomes occurring later than 6 months in our analysis was justified. Some evidence suggests that Xpert might reduce time to multidrug-resistant tuberculosis treatment by around 3 weeks.^12^ A 2017 retrospective cohort study^13^ of the pathways to treatment of patients with multidrug-resistant tuberculosis (done at the same time as XTEND) suggests a reduction in the time to treatment of around 17 days; however, the authors found no difference in the proportions of patients who initiated treatment before 6 months, between patients diagnosed with Xpert compared with patients diagnosed with other methods, and did not follow-up the cohort to measure outcomes. Because of the inconclusive nature of this evidence and because we aimed to assess real-world cost-effectiveness with an empirical approach, we did not include any additional effect on multidrug-resistant tuberculosis in our estimates.

We were guided in our analytical approach by the reference case developed by the Methods for Economic Evaluation Project (MEEP).^14^ A full methodological description of the study according to the MEEP reference case can be found in the appendix. Our approach also reflects the economic school of thought that, even when trial results find no significant evidence of an effect, a full cost-effectiveness analysis should be done, presenting all uncertainty in both costs and effects^15^ before concluding on cost-effectiveness.

We did comparative cost and cost-effectiveness analyses of Xpert versus the standard of care, sputum smear microscopy. From a societal perspective, we compared the cost per person investigated for tuberculosis and the cost per disability-adjusted life-year (DALY) averted. The population studied was a cohort of people being assessed for tuberculosis and attending primary health-care clinics in South Africa.

The study was approved by the research ethics committees of the University of Cape Town, University of the Witwatersrand, London School of Hygiene & Tropical Medicine, and WHO. Health department officials and facility managers provided permission to do the study in the selected facilities and written informed consent was obtained from respondents.

### Study intervention and comparator

We compared the intervention, Xpert MTB/RIF, with the comparator, sputum smear microscopy, as initial tests for tuberculosis. However, both intervention and comparator required additional technologies for their implementation.^16^ Xpert required changes to the tuberculosis diagnostic and treatment pathways. Because Xpert provides a signal for rifampicin resistance, the retreatment tuberculosis regimen for patients who did not respond to first-line treatment or who have previously had tuberculosis treatment is no longer required. A figure of the sputum smear microscopy and Xpert algorithms studied is contained in the appendix. Additionally, the roll-out of Xpert required supportive activities, including restructuring of workloads in laboratories and quality control.^17^ Xpert roll-out commenced in March, 2011, with sequential capacitation of laboratories with large, medium, and small sputum specimen volumes. National roll-out was completed in December, 2013.

### Data collection

In view of the dearth of primary cost data available on tuberculosis services in South Africa,^18^ we collected extensive primary data on outcomes, patient events (or resource use), and costs. We used this primary data to construct a dataset containing individual costs and outcomes for every XTEND participant as the basis for our analysis.

Because the details of our costing methods, prices, and unit costs of each patient event are reported elsewhere,^17^, ^19^ we only provide a brief overview in this Article. We used primary data collection methods to estimate all costs (including human resource costs) at both the site and above-site levels. We included all capital and recurrent costs at the facility and laboratory (site) level, and the above-site level costs incurred by the central laboratory services, including quality control and training for both Xpert and sputum smear microscopy.^17^ We collected cost data from all 20 XTEND peripheral laboratories and one reference laboratory. We measured diagnostic test costs at each of these laboratories during two periods (in the first 2 months and last 2 months of the XTEND study).

We estimated the cost of drug-sensitive tuberculosis treatment from ten XTEND study clinics purposively selected to represent the range in number of annual primary health-care visits (size of clinic) and geography (urban, rural, and peri-urban) of primary health-care clinics in South Africa. We primarily used bottom-up methods to cost visits, using interviews, facility records, and observations. For multidrug-resistant tuberculosis treatment costs, we retrospectively collected patient-level and clinic-level cost data from five non-XTEND study health facilities: two primary care clinics, one community health centre, one local subacute inpatient facility, and a central tuberculosis hospital in Cape Town.^19^ We estimated the costs of both centralised and decentralised care models. Finally, we sourced HIV costs from a systematic review.^20^ A summary of the provider unit costs used is presented in the appendix, together with a list of the publications containing further details of the primary cost data.

We estimated the direct and indirect costs incurred by study participants while accessing care from the reported start of tuberculosis symptoms.^21^, ^22^ In summary, three samples of patients were interviewed.^21^ We consecutively subsampled one in three of the patients treated at the ten XTEND study clinics selected for health service costing and succeeded in enrolling a cohort of 351 individuals being investigated for tuberculosis, who were interviewed at XTEND enrolment and then at follow-up 6 months later. We interviewed a further cohort of 168 patients on first-line tuberculosis treatment, who were not part of the XTEND cohort (to protect from interview overload), at 2 months and 5 months from start of treatment. Finally, we interviewed a cross-section of 134 patients with multidrug-resistant tuberculosis from Western Cape.^22^ All respondents were asked about their health-seeking activities, the associated costs, income loss, the time cost of carers, and any coping strategies used.

We estimated total costs for the Xpert and the sputum smear microscopy groups by multiplying unit or per-event costs by the number of health service use events reported in the XTEND study cohort. Data on patient events (outpatient visits, treatment regimens, and diagnostic tests used) were collected from case note abstractions (from paper and electronic records) and by self-report from the XTEND participant follow-up interviews (done with all XTEND participants). Where discrepancies between data sources were found, a set of decision rules were developed and applied on the basis of plausibility and consistency between data.

### Outcomes

We estimated mean total costs and cost per DALY averted. DALYs averted were the main measure of incremental outcome. We used deaths reported from the XTEND study (appendix) to estimate years of life lost. The XTEND study's primary outcome was mortality among participants investigated for tuberculosis, measured 6 months after enrolment. The XTEND study found no evidence of a difference in mortality risk (91 [4%] of 2324 *vs* 116 [5%] of 2332; adjusted risk ratio 1·10, 95% CI 0·75–1·62).^11^ We estimated the years lived with disability of its participants using the number of days with tuberculosis symptoms, assuming symptoms either stopped 2 weeks into treatment, continued to death, or stopped within 2 weeks of not returning to care. We applied disability weights used in the Global Burden of Disease 2010.^23^ For people living with HIV/AIDS on antiretroviral therapy for HIV, we applied the tuberculosis only disability weight.^23^ We estimated all DALYs averted using a 3% discount rate and no age weighting.

### Statistical analysis

To complete our patient-level dataset, we first had to address the missing data by design (due to subsampling) for the patient-incurred costs. We used two methods: applying mean per-event costs from our subsample and applying multiple imputation to assign per-event costs from our subsample of 351 to the total sample of 4656 XTEND participants.^21^ The multiple imputation covariates included sex (binary), number of years at school (ordinal), self-reported HIV status (categorical), initial tuberculosis test result (binary), geographical location (binary: urban or rural), income (categorical), number of days with at least one symptom (continuous), anyone else in household with a regular job (binary), household socioeconomic status (categorical: derived through principal component analysis), number of health-care visits (continuous), country of birth (categorical), age (continuous), province (categorical), and distance from residence to clinic (ordinal). In view of the high number of zero observations in our subsample for some per-event costs, we applied a two-part model, which first predicts non-zero costs for each type of patient event, and then predicts the level of costs for events with a non-zero cost.

The cost and cost-effectiveness analyses reflected the clustered design and baseline imbalance between study groups.^24^ For both analyses, we selected a two-stage cluster-level analysis with non-parametric two-stage bootstrapping.^25^ The two-stage bootstrapping algorithm performs resampling with replacement in two stages. In the first stage, it resamples clusters and in the second stage it resamples individuals within clusters. A shrinkage estimator is used to correct for potential overestimation of variance. Bootstrap datasets are then constructed, combining the resampled shrunken cluster means with resampled individual-level residuals.

For the cost analysis, we applied a multiple ordinary least square regression model with robust standard errors using 200 bootstrap replications and adjusting for HIV status, socioeconomic status, ethnicity, education, marital status, age group, sex, province, body-mass index group, and number of symptoms. We calculated the predicted cost for each patient and subsequently collapsed the data across cluster and calculated the residual (difference of observed and predicted) costs for each cluster. In the second stage, we ran an ordinary least squares regression analysis at the cluster level to assess differences in costs between Xpert and sputum smear microscopy groups using the residuals as the dependent variable. We used this method to estimate incremental costs between the two study groups.

For our cost-effectiveness analysis, we sampled (with two-stage bootstrapping) from adjusted costs (produced in the cost analysis) and adjusted deaths (using the adjusted analysis on outcomes as outlined in the main trial paper; appendix)^24^ to estimate cost-effectiveness ratios per death and DALYs averted, running 10 000 replications (appendix). We present the uncertainty around our incremental cost-effectiveness ratios with cost-effectiveness planes and acceptability curves and apply a one-way sensitivity analysis around 0% and 3% discount rates. All costs are presented in 2014 US$.

We used Stata 14.1 for the statistical analysis and prepared the dataset linking events and costs in Excel 2007.

### Role of the funding source

The funder of the study had no role in study design, data collection, data analysis, data interpretation, or writing of the report. The corresponding author had full access to all the data in the study and had final responsibility for the decision to submit for publication.

## Results

We stratified summary data of patient events by subgroups of participants: participants with an initial positive result; participants with an initial negative result, with no subsequent diagnostic test; and participants with an initial negative result, but received follow-up diagnostic tests (table 1). Although the Xpert group had more initial positive tuberculosis results than sputum smear microscopy (200 [7%] of 2324 patients *vs* 174 [7%] of 2332), fewer patients had follow-up tests (211 [9%] of 2324 patients *vs* 441 [19%] of 2332). Total treatment initiations were lower in the Xpert group than the sputum smear microscopy group (250 events in 2324 patients *vs* 291 events in 2332 patients). This included lower numbers of patients initiated on a retreatment regimen (12 events in 2324 patients *vs* 34 events in 2332 patients). The total number of hospital days was higher in the Xpert group (546 days *vs* 506 days), primarily incurred by participants with multidrug-resistant tuberculosis. The total number of cultures done in the Xpert group was also lower (167 tests *vs* 497 tests), but the total number of antibiotic courses provided was higher (659 *vs* 462). The total number of HIV tests done was similar in both groups, as was the total number of patients starting antiretroviral therapy.

**Table 1:** Total patient events reported by study group and initial tuberculosis test results

		**Sputum smear microscopy group (n=2332)**					**Xpert group (n=2324)**				
		Initial positive tuberculosis test result	Initial negative tuberculosis test result, followed up with radiograph or culture	Initial negative tuberculosis test result with no follow-up with radiograph or culture	Initial tuberculosis test result unknown	Total	Initial positive tuberculosis test result	Initial negative tuberculosis test result, followed up with radiograph or culture	Initial negative tuberculosis test result with no follow-up with radiograph or culture	Initial tuberculosis test result unknown	Total
People investigated for tuberculosis		174	441	1645	72	2332	200	211	1782	131	2324
Patient events											
	Total nights in hospital	47 (9%)	347 (69%)	90 (18%)	22 (4%)	506 (100%)	170 (31%)	281 (51%)	42 (8%)	53 (10%)	546 (100%)
	Patients on ART before enrolment	26 (9%)	73 (27%)	165 (60%)	11 (4%)	275 (100%)	18 (6%)	54 (18%)	201 (69%)	19 (7%)	292 (100%)
	Patients starting ART after enrolment	35 (12%)	73 (26%)	162 (58%)	11 (4%)	281 (100%)	50 (20%)	30 (12%)	161 (64%)	11 (4%)	252 (100%)
	Total health-care visits for tuberculosis symptoms	194 (9%)	492 (22%)	1430 (64%)	118 (5%)	2234 (100%)	315 (11%)	358 (13%)	1991 (71%)	151 (5%)	2815 (100%)
Treatment											
	Total treatment initiations	160 (55%)	70 (24%)	43 (15%)	18 (6%)	291 (100%)	180 (72%)	33 (13%)	21 (8%)	16 (6%)	250 (100%)
	Category 1	134 (55%)	61 (25%)	36 (15%)	14 (6%)	245 (100%)	161 (72%)	31 (14%)	17 (8%)	15 (7%)	224 (100%)
	Category 2 (retreatment)	19 (56%)	8 (24%)	3 (9%)	4 (12%)	34 (100%)	9 (75%)	2 (17%)	1 (8%)	0	12 (100%)
	Multidrug resistant tuberculosis treatment	1 (33%)	1 (33%)	1 (33%)	0	3 (100%)	2 (100%)	0	0	0	2 (100%)
	Other	3 (50%)	1 (17%)	2 (33%)	0	6 (100%)	4 (67%)	0	2 (33%)	0	6 (100%)
	Missing	4 (80%)	0	1 (20%)	0	5 (100%)	7 (78%)	0	1 (11%)	1 (11%)	9 (100%)
Diagnostic and monitoring tests											
	Total chest radiographs	17 (13%)	104 (76%)	0	15 (11%)	136 (100%)	13 (9%)	116 (82%)	0	13 (9%)	142 (100%)
	Total antibiotic courses	22 (5%)	107 (23%)	307 (66%)	26 (6%)	462 (100%)	76 (12%)	96 (15%)	436 (66%)	51 (8%)	659 (100%)
	Total HIV tests	49 (8%)	132 (22%)	376 (63%)	37 (6%)	594 (100%)	78 (13%)	74 (13%)	392 (67%)	43 (7%)	587 (100%)
	Total sputum smear microscopy tests (excluding initial test)	204 (18%)	226 (20%)	677 (59%)	35 (3%)	1142 (100%)	252 (78%)	33 (10%)	19 (6%)	19 (6%)	323 (100%)
	Total Xpert tests (excluding initial test)	0	0	0	0	0	22 (17%)	26 (20%)	52 (40%)	31 (24%)	131 (100%)
	Total cultures	82 (16%)	388 (78%)	0	27 (5%)	497 (100%)	24 (14%)	136 (81%)	0	7 (4%)	167 (100%)
	Total line probe assays	69 (55%)	29 (23%)	25 (20%)	2 (2%)	125 (100%)	73 (61%)	19 (16%)	22 (18%)	5 (4%)	119 (100%)
	Total drug susceptibility tests	43 (61%)	13 (19%)	10 (14%)	4 (6%)	70 (100%)	30 (50%)	10 (17%)	17 (28%)	3 (5%)	60 (100%)

ART=antiretroviral therapy.

Applying the unit costs to patient events, we found the mean total cost per participant incurred was US$312·58 (95% CI 252·46–372·70) in the Xpert group and $298·58 (246·35–350·82) in the sputum smear microscopy group (table 2). The mean health service (provider) cost per participant was $168·79 (149·16–188·42) for Xpert and $160·46 (143·24–177·68) for the sputum smear microscopy. We found that Xpert increases the cost of initial diagnostic tests, but this was mitigated by a decrease in the costs of follow-on diagnostics and treatment. A small difference was reported in total mean patient-incurred costs between the Xpert and the sputum smear microscopy groups ($143·79 *vs* $138·12). After adjustment for baseline imbalance between the two groups, we found no significant difference in costs between Xpert and the sputum smear microscopy groups (table 3). Only health service diagnostic costs were found to differ significantly between the two groups, with Xpert costs being higher than sputum smear microscopy (p=0·023).

**Table 2:** Total and mean costs by study group

	**Sputum smear microscopy group (n=2332)**		**Xpert group (n=2324)**	
	Total cohort cost ($)	Mean cost per cohort member ($; 95% CI)	Total cohort cost ($)	Mean cost per cohort member ($; 95% CI)
**Health service costs**				
Hospital treatment	24 131	10·35 (4·08–16·62)	20 765	8·94 (2·66–15·21)
Health service visits	19 283	8·27 (6·84–9·70)	24 298	10·46 (7·00–13·91)
Antibiotics	128	0·06 (0·05–0·06)	183	0·08 (0·07–0·08)
Other treatment of symptoms	6418	2·75 (2·31–3·20)	8398	3·61 (3·05–4·18)
Sputum smear microscopy (for diagnosis)	20 222	8·67 (8·62–8·72)	0	0·00
Xpert	0	0·00	56 758	24·42 (24·30–24·54)
Chest radiograph	1492	0·64 (0·51–0·77)	1558	0·67 (0·52–0·82)
Culture	9354	4·01 (3·65–4·38)	2528	1·09 (0·90–1·27)
Line probe assay	3182	1·36 (0·88–1·84)	1408	0·61 (0·21–1·00)
Drug-susceptibility testing	3759	1·61 (0·95–2·27)	3222	1·39 (0·88–1·90)
Subtotal diagnosis	87 969	37·72 (31·03–44·41)	119 118	51·26 (43·79–58·72)
Category 1 treatment	42 757	18·33 (16·15–20·52)	40 409	17·39 (15·25–19·52)
Category 2 retreatment	8823	3·78 (2·51–5·06)	3318	1·43 (0·62–2·24)
Multidrug resistant-tuberculosis treatment	37 263	15·98 (2·71–29·25)	37 668	16·21 (2·90–29·52)
Subtotal treatment	88 844	38·10 (24·02–52·17)	81 396	35·02 (20·93–49·12)
Subtotal HIV testing and treatment	197 389	84·64 (78·65–90·64)	191 758	82·51 (76·62–88·40)
Total health service cost	374 202	160·46 (143·24–177·68)	392 271	168·79 (149·16–188·42)
**Patient incurred cost**				
Diagnosis	244 520	104·85 (63·03–146·68)	267 454	115·08 (70·43–159·74)
Treatment	55 119	23·64 (20·79–26·48)	46 249	19·90 (17·30–22·50)
HIV testing and treatment	22 464	9·63 (9·06–10·20)	20 471	8·81 (8·27–9·35)
Total patient incurred cost	322 103	138·12 (95·98–180·27)	334 174	143·79 (98·30–189·28)
**Total cost**				
Total cost	696 306	298·58 (246·35–350·82)	726 445	312·58 (252·46–372·70)

Costs are in US$ for 2014.

**Table 3:** Mean incremental costs of Xpert versus sputum smear microscopy per cohort member

	Simple imputation dataset*		Multiple imputation dataset*	
	β coefficient† (95% CI)	p value	β coefficient† (95% CI)	p value
**Incremental difference in total costs per cohort member**				
Xpert *vs* sputum smear microscopy	23·34 (−22·86 to 69·55)	0·303	29·63 (−48·98 to 108·24)	0·439
Constant	−11·52 (−44·20 to 21·15)	0·468	−14·56 (−70·14 to 41·03)	0·589
**Incremental difference in patient incurred costs per cohort member**				
Xpert *vs* sputum smear microscopy	9·30 (−23·33 to 41·94)	0·557	15·59 (−50·29 to 81·47)	0·625
Constant	−4·61 (−27·68 to 18·47)	0·680	−7·64 (−54·23 to 38·95)	0·734
**Incremental difference in provider costs per cohort member**				
Xpert *vs* sputum smear microscopy	14·04 (−11·91 to 39·99)	0·271	14·04 (−11·91 to 39·99)	0·271
Constant	−6·92 (−25·27 to 11·44)	0·439	−6·92 (−25·27 to 11·44)	0·439
**Incremental difference in provider costs (diagnostic test only) per cohort member**				
Xpert *vs* sputum smear microscopy	14·65 (2·30 to 27·01)	0·023	14·65 (2·30 to 27·01)	0·023
Constant	−7·31 (−16·04 to 1·43)	0·096	−7·31 (−16·04 to 1·43)	0·096

Costs are in US$ for 2014.

* Simple imputation using means of patient costs from subsample. Both datasets adjusted for HIV status, socioeconomic status quintiles (based on principal component analysis), ethnicity, education, marital status, age group, sex, province, body-mass index group, and number of symptoms.

† We have the general form of a regression equation: Y=constant + (βX). Where Y is the difference in costs between the Xpert and sputum smear microscopy groups (ie the incremental cost) and X is a binary variable that represents the groups (Xpert=1 and sputum smear microscopy=0). For example, in the case of total costs, the incremental cost between Xpert and sputum smear microscopy is −11·52 + 23·34=11·82.

**Figure 1:**
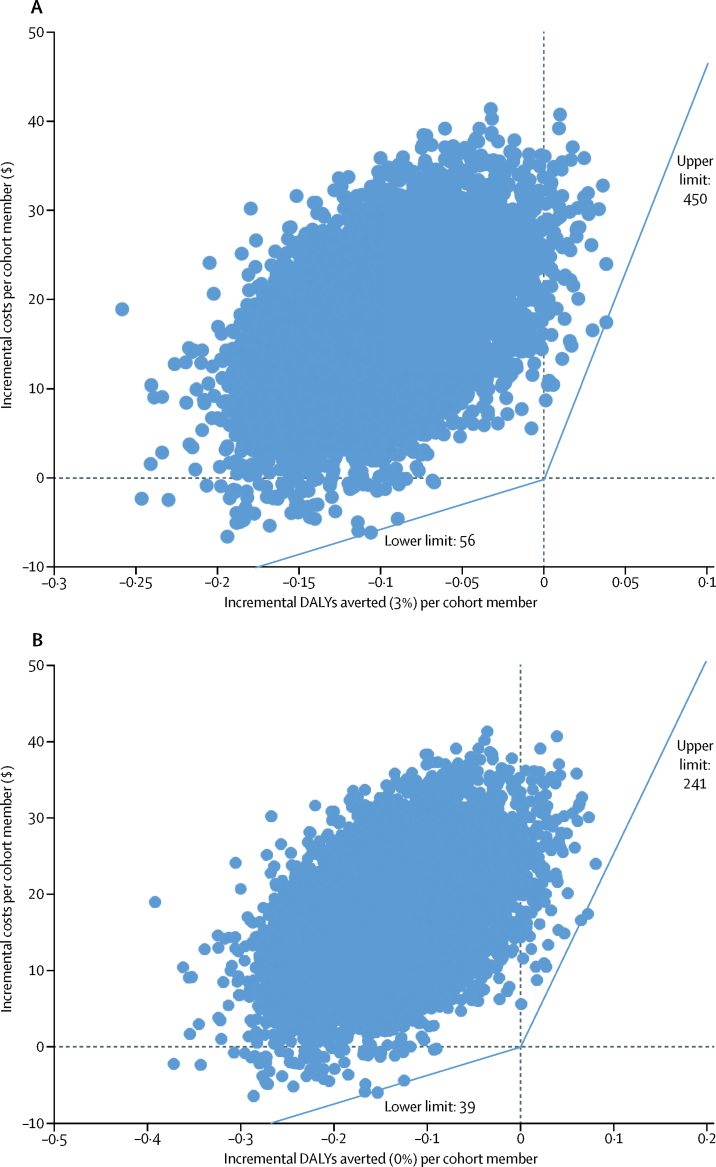
Cost-effectiveness planes of Xpert versus sputum smear microscopy Cost-effectivness planes showing the results of two different models of non-parametric two-stage bootstrapping using 10 000 replications to produce incremental costs per DALYs averted for Xpert versus sputum smear microscopy, with 3% (A) and 0% (B) discount rates. The x-axis represents the difference in DALYs averted, and the y-axis represents the difference in costs for the imputed data using 10 000 replications. Each point in the scatterplot represents one bootstrap iteration. Costs are in 2014 US$. DALY=disability-adjusted life-year.

**Figure 2:**
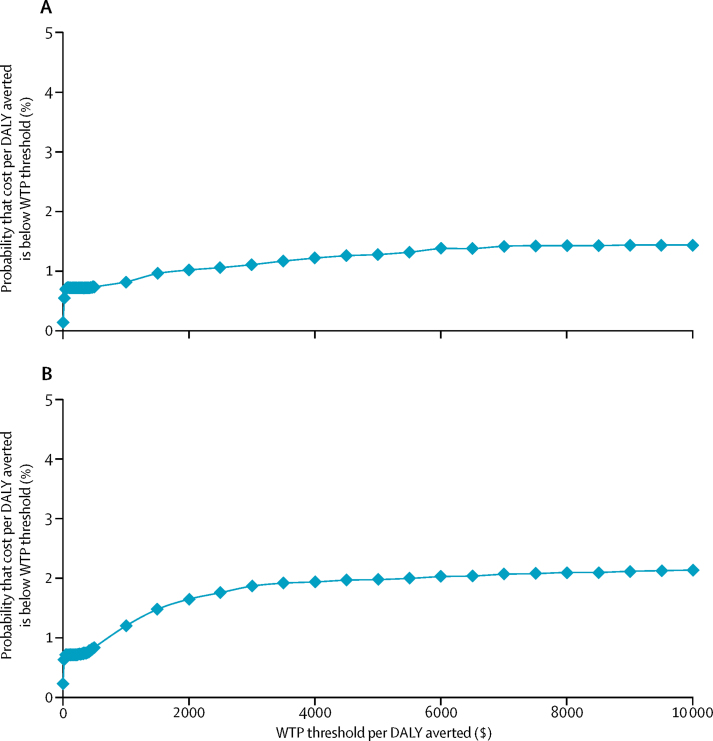
Cost-effectiveness acceptability curves of Xpert versus sputum smear microscopy The cost-effectiveness acceptability curves were generated by running 10 000 replications. The probability of Xpert being cost-effective was examined at different WTP thresholds, with 3% (A) and 0% (B) discount rates. Costs are in 2014 US$. WTP=willingness to pay. DALY=disability-adjusted life-year.

The mean incremental costs and mean incremental effect of Xpert compared with sputum smear microscopy fall into the upper left quadrant of the cost-effectiveness plane (figure 1, table 4). This was the same if we used a 0% or 3% discount rate. After application of a wide range of willingness to pay thresholds, our results suggest that there is less than 3% probability that Xpert is cost-effective in this population (figure 2).

**Table 4:** Incremental costs and outcomes of Xpert versus sputum smear microscopy

	Simple imputation dataset*			**Multiple imputation dataset**†		
	Deaths averted	DALYs averted (0%)	DALYs averted (3%)	Deaths averted	DALYs averted (0%)	DALYs averted (3%)
Incremental difference in outcomes (95% CI)	−0·011 (−0·028 to 0·01)	−0·13 (−0·68 to 0·42)	−0·09 (−0·42 to 0·26)	−0·011 (−0·028 to 0·01)	−0·13 (−0·68 to 0·42)	−0·09 (−0·42 to 0·26)
Mean ICER‡	−1148	−92	−129	−1534	−123	−172

Costs are in US$ for 2014. DALY=disability-adjusted life-year. ICER=incremental cost-effectiveness ratio.

* Incremental difference in cost $12·25 (95% CI −47·10 to 69·18).

† Incremental difference in cost $16·36 (95% CI −70·07 to 104·58).

‡ ICERs are negative and therefore cannot be used to assess cost-effectiveness; see figures 1 and 2.

## Discussion

We found that the Xpert roll-out in South Africa, for a population of presumptive drug-susceptible tuberculosis participants, did not decrease or increase costs of tuberculosis investigation, diagnosis, and treatment (in the period from symptoms to 6 months after initial investigation), and, in this sense, can be described as cost-neutral. The additional cost of Xpert equipment and tests was mitigated by a reduction in costs elsewhere in the tuberculosis cascade of care. In the Xpert group, fewer people started treatment on an empirical basis and began retreatment. However, Xpert showed higher diagnostic costs than sputum smear microscopy, including higher levels of prescriptions of other antibiotics and additional symptom seeking visits, possibly due to lower levels of empirical treatment for tuberculosis. Combining cost neutrality with the absence of any significant mortality effect, we found that the introduction of Xpert did not improve the cost-effectiveness of drug-susceptible tuberculosis diagnosis and treatment in South Africa during the early stages of the national Xpert roll-out.

This finding differs from modelled predictions of the cost-effectiveness of Xpert introduction. Our previous model,^5^ and many others, assumed that for people investigated for tuberculosis with an initial negative tuberculosis test result the follow-on diagnostic pathway would either be highly sensitive, but with low specificity (for example, a chest radiography or high levels of empirical treatment) or use a diagnostic such as culture, but limited to a small proportion of patients.^5^ Sensitivity and specificity of this pathway were therefore assumed to be negatively correlated with one another.^5^ By reducing the numbers of false-negative tuberculosis cases moving along this diagnostic pathway, Xpert would either reduce overtreatment or prevent mortality.

The TB-NEAT trial^26^ found that Xpert did not affect mortality and could replace, but not reduce, empirical treatment. The absence of the anticipated reduction in empirical treatment might have been partly attributable to providers becoming more aware of the limitations of tuberculosis diagnostics generally.^27^ Likewise, XTEND found no effect on mortality, and a reduction in follow-up along the initial test negative pathway during roll-out.^11^ In a setting with the momentum of a national roll-out, these reductions in follow-up tests could be attributable to clinicians overly trusting Xpert results, as the new technology is promoted. This explanation is supported by the findings of an XTEND substudy^28^ examining the pathways of participants with initial negative results, which found much lower levels of algorithm adherence and HIV testing among participants with an initial Xpert test compared with participants with sputum smear microscopy.

Additionally, both of the study groups in XTEND had high levels of loss to follow-up and moderate levels of antiretroviral therapy coverage, which potentially mitigated any effect on outcomes that would result from a correct tuberculosis diagnosis.^11^ Similar findings were seen in Brazil, where high levels of loss to follow-up undermined any potential increment effect of Xpert.^29^ Therefore, in the case of XTEND, TB-NEAT, and the Brazil study, the cost and cost-effectiveness of Xpert were fundamentally affected by both the existing standard-of-care comparator, and the way in which Xpert was implemented, and the adherence along both the tuberculosis and HIV diagnostic and treatment pathways, rather than the performance of the technology itself.

Prior to WHO guideline recommendations and roll-out, economic evaluations of tuberculosis diagnostics were not able to access data to adequately parameterise standard-of-care clinical practice, or the algorithms used, and were not able to predict how these practices might change with different approaches to implementation. The extensive primary data collection permitted by a phased approach to roll-out enabled us to correct our initial estimates. More broadly, our research, together with the emerging research on malaria diagnostics, highlights the importance of post-hoc real-world economic analyses of new technologies to validate initial predictions.^16^ Additionally, our findings provide an example to support the broader calls for model-based economic evaluations to formally consider uncertainty (and the additional costs) of implementation of new diagnostic technologies in general.^30^

Our study has several limitations. First, the methods to account for clustering and imbalance can generate narrower CIs than with other more complex and time-consuming methods such as multilevel modelling, even if they produce unbiased estimates of the mean incremental cost-effectiveness ratio.^24^ This means that our results can seem more certain than is the case. However, considering our probability of cost-effectiveness is extremely low, this underestimation of uncertainty is unlikely to affect our conclusion. Second, although we imputed patient-incurred costs, and captured some uncertainty in this imputation, our sample of primary cost data was not powered to detect true differences in patient-incurred costs between Xpert and sputum smear microscopy. Third, we had to make crude assumptions about duration of symptoms to estimate years lived with disability. However, the level of years lived with disability is dwarfed by years of life lost in the DALY calculations.

Fourth, we limited the timeframe of our assessment to a within-trial period. This potentially excludes potential benefits for participants with multidrug-resistant tuberculosis and future relapses. We take this approach because little real-world evidence exists to show an effect of Xpert on the costs or outcomes of people with multidrug-resistant tuberculosis or on tuberculosis relapses in South Africa. As with the diagnosis of drug-susceptible tuberculosis, outcomes are likely to also be affected by the follow-on treatment pathway. However, Xpert might conceivably have a positive effect on multidrug-resistant tuberculosis outcomes and transmission through the reduction of time to treatment. Conversely, some additional costs might also occur due to people with multidrug-resistant tuberculosis being placed on treatment without comprehensive drug susceptibility testing. However, not enough data exist to investigate either hypothesis. We therefore limit our findings and conclusions to people with drug-susceptible tuberculosis.

Fifth, generally within-trial economic evaluations might be seen as limited because they might not represent the full range of evidence available (unlike modelled evaluation, which involves the synthesis of all data). However, our study is a multicentre cluster randomised study of 40 sites in South Africa in addition to being the only robust and comprehensive source of real-world cost and outcomes data available during the period of the early Xpert roll-out in South Africa. That said, even pragmatic trials do not fully capture real-world practice. Routine data systems could be an additional data source if of sufficient quality and appropriately analysed. Analysts in other settings with less data scarcity should continue to consider all relevant evidence and continue to do model-based economic evaluations of tuberculosis diagnostics as the preferential approach.

Although our results suggest that South Africa might not have made a sound investment in Xpert initially, this does not mean that Xpert will not be cost-effective in other settings, or in South Africa in the future. Our results instead suggest that policy makers need to consider the context that Xpert (and other tuberculosis diagnostics) are placed in before providing large-scale investment. Our research highlights the need to simultaneously consider and define complementary interventions to optimise the Xpert-based tuberculosis diagnostic and treatment pathway. Finally, our study shows the importance for global policy makers and funders (and the analysts supporting them) to consider the uncertainty caused by implementation constraints when making recommendations for guidelines and investing in new tuberculosis diagnostics.
