# Supplementary material for: Cost-effectiveness of Xpert MTB/RIF for tuberculosis diagnosis in South Africa: a real-world cost analysis and economic evaluation
Source: Lancet Glob Health. 2017 Jun 12;5(7):e710–9. doi: 10.1016/S2214-109X(17)30205-X (PMC5471605; doi:10.1016/S2214-109X(17)30205-X)
Supplement: Supplementary appendix [file mmc1.pdf]

THE LANCET
Global Health

Supplementary appendix

This appendix formed part of the original submission and has been peer reviewed. We post it as supplied by the authors.

Supplement to: Vassall A, Siapka M, Foster N, et al. Cost-effectiveness of Xpert MTB/RIF for tuberculosis diagnosis in South Africa: a real-world cost analysis and economic evaluation. *Lancet Glob Health* 2017; **5:** e710–19.

SUPPLEMENTARY MATERIALS

Table S1: Baseline characteristics by study arm (n=4656)

			**Xpert (n=2324)**	**Microscopy (n=2332)**
Age	In years	median (IQR)	35 (28-45)	37 (29-48)
Sex	Female	% (n)	64·2% (1493)	59·9% (1398)
Country of birth	South Africa	% (n)	92·4% (2148)	95·1% (2218)
Marital status	Single	% (n)	53·1% (1233)	56·0% (1305)
Main income source	Employed	% (n)	41·6% (968)	46·9% (1093)
	Govt. grant	% (n)	20·9% (485)	15·6% (363)
	Other	% (n)	37·5% (871)	37·6% (876)
Dwelling	House	% (n)	52·1% (1211)	66·8 % (1557)
	Traditional	% (n)	25·9% (601)	16·2% (378)
	Other	% (n)	22·0% (512)	17·0% (397)
HIV status, self-report	Negative	% (n)	27·5% (639)	29·9% (697)
	Positive[1]	% (n)	45·3% (1052)	49·5% (1154)
	Unknown	% (n)	27·2% (633)	20·6% (481)
CD4 count (self-report)		median (IQR)	303 (171-457)	315 (192-480)
		n	521	609
Ever taken ART (among HIV+)	Yes	% (n/N)	33·5% (352/1052)	32·7% (377/1154)
Time on ART	Years	median (IQR)	1·8 (0·7-3·7)	1·2 (0·4-3·0)
Previous tuberculosis	Yes	% (n)	14·8% (344)	16.0% (374)
Body mass index (kg/m^2)	<18·5	% (n)	8·7% (202)	12·4% (288)
	18·5-25	% (n)	45·7% (1062)	46·2% (1077)
	>25	% (n)	45·6% (1060)	41·5% (967)
Karnofsky score	≤80	% (n)	41·3% (960)	42·1% (981)
	90	% (n)	40·5% (941)	45·2% (1055)
	100	% (n)	18·2% (423)	12·7% (296)
<u>Symptoms reported</u>				
Cough	Yes	% (n)	80·9% (1879)	83·7% (1952)
Night sweats	Yes	% (n)	40·7% (946)	49·4% (1152)
Weight loss	Yes	% (n)	44·3% (1030)	51·9% (1210)
Fever	Yes	% (n)	38·1% (886)	51·7% (1206)
Total number of symptoms[2]	0	% (n)	9·8% (227)	6·0% (139)
	1	% (n)	23·5% (547)	19·6% (456)
	2	% (n)	32·4% (752)	27·0% (628)
	≥3	% (n)	34·3% (798)	47·5% (1109)
Main reason for visit	TB symptoms	% (n)	72·7% (1689)	74·4% (1735)
	HIV care	% (n)	8·3% (194)	9·2% (215)
	HIV test	% (n)	6·0% (140)	6·4% (150)
	General/chronic	% (n)	7·1% (164)	6·8% (158)
	ANC	% (n)	5·4% (126)	2·1% (50)
	Other	% (n)	0·5% (11)	1·0% (24)

Data are n (%) or median (IQR) or n/N (%) unless otherwise stated.
ANC=antenatal clinic attendance; ART=antiretroviral therapy; IQR=Inter-quartile range; TB=tuberculosis.
[1] Percentage HIV positive out of those with known HIV status 62.2% (1052/1691) and 62.3% (1154/1851) in the Xpert and microscopy arms, respectively; [2]Number of symptoms based on current cough, night sweats, fever and weight loss.

Table S2 – Unit (provider) cost of patient events and resources (US$2014)

	Provider cost	
**Variable**	**Mean**	**SD**
Category 1 treatment episode	171.12	28.33
Category 2 treatment (retreatment) episode	252.95	64.37
MDR treatment episode	6,244.31	1,677.45
Per visit cost	12.54	66.47
Per Xpert	24.42	2.97
Per X-ray	15.17	7.74
Per course of antibiotics	0.3	0.09
Per microscopy	8.67	1.19
Per culture	19.57	8.6

Full details of all costs can be found in:

1: Cunnama L, Sinanovic E, Ramma L, Foster N, Berrie L, Stevens W, Molapo S, Marokane P, McCarthy K, Churchyard G, Vassall A. Using top-down and bottom-up costing approaches in LMICs: the case for using both to assess the incremental costs of new technologies at scale. *Health Econ* 2016; **25** (suppl 1)**:** 53–66.
2: Ramma L, Cox H, Wilkinson L, Foster N, Cunnama L, Vassall A, Sinanovic E. Patients' costs associated with seeking and accessing treatment for drug-resistant tuberculosis in South Africa. *Int J Tuberc Lung Dis* 2015; **19:** 1513–19.
3: Foster N, Vassall A, Cleary S, Cunnama L, Churchyard G, Sinanovic E. The economic burden of TB diagnosis and treatment in South Africa. *Social Sci Med* 2015; **130:** 42–50.
4: Sinanovic E, Ramma L, Vassall A, Azevedo V, Wilkinson L, Ndjeka N, McCarthy K, Churchyard G, Cox H. Impact of reduced hospitalisation on the cost of treatment for drug-resistant tuberculosis in South Africa. *Int J Tuberc Lung Dis* 2015; **19:** 172–78.

Table S3: Effect of Xpert MTB/RIF on the primary and secondary outcomes

**Primary outcome**	**Xpert**		**Microscopy**		**Risk ratio (95% CI), p-value**	
	**Deaths/N**	**%***	**Deaths/N**	**%***	**Unadjusted**	**Adjusted[1]**
Mortality risk over 6 months (n=4656)	91/2324	3·9%	116/2332	5·0%	0·86 (0·56-1·28), p=0·43	1·10 (0·75-1·62), p=0·61
**Secondary outcomes**	**Xpert MTB/RIF**		**Microscopy**		**Ratio measure (95% CI), p-value**	
	**n/N**	**%***	**n/N**	**%***	**Unadjusted**	**Adjusted**
Proportion test positive (n=4411)	200/2176	9·2%	174/2235	7·8%	1·27 (0·81-2·00), p=0·27	1·49 (1·00, 2·23)[2], p=0·05
Initial loss to follow-up (n=374)[3]	34/200	17·0%	26/174	14·9%	0·97 (0·48-1·96), p=0·93	0·96 (0·48-1·93)[4], p=0·91
Proportion treated for TB (n=4656)	250/2324	10·8%	291/2332	12·5%	0·88 (0·60-1·29), p=0·48	1·04 (0·76-1·43)[2], p=0·79
Proportion microbiologically confirmed (n=541)	196/250	78·4%	189/291	65·0%	1·21 (0·99-1·47), p=0·06	1·20 (0·98-1·47)[2], p=0·07

CI=confidence interval; *summary ignores cluster

[1]adjusted for age group, sex, body mass index group, number of tuberculosis symptoms and HIV status; [2]adjusted for age group, sex, body mass index group and number of tuberculosis symptoms; [3]due to zero events in two clusters, one event has been added to all clusters, [4]adjusted for body mass index group and number of tuberculosis symptoms; TB=tuberculosis

Table S4 - Summary of methodological choices based MEEP Reference Case

The Reference Case Reporting Template

Introduction and background

**Principle 1: An economic evaluation should be communicated clearly and transparently to enable the decision maker(s) to interpret the methods and results**
**Fully and accurately describe the key elements of the decision problem:**<br><br>Population (description and characteristics, see also principle 8:<br><br>Presumptive Cases of Tuberculosis in South Africa identified through passive screening<br><br>Intervention(s): Xpert MTB/RIF as the initial diagnostic test for Tuberculosis<br><br>Comparator(s) (see principle 2): Smear microscopy as the initial diagnostic test for Tuberculosis (current 'best practice in routine use')<br><br>Measure(s) of outcome (see principle 4): Disability Adjusted Life Years (DALYs)<br><br>**Link to policy**<br>Intended user of the research output: National Department of Health (NDOH), South Africa, other countries in sub-Saharan Africa and global funding agencies such as the Global Fund for Aids, Tuberculosis and Malaria<br>Relevance of the economic evaluation to health practice and policy decisions: The NDOH, South Africa, was an early adopter of Xpert MTB/RIF. A detailed evaluation of the roll-out of Xpert MTB/RIF can help other countries decide on whether to also invest in Xpert MTB/RIF; and assist the NDOH learn lessons to support Xpert MTB/RIF and the introduction of other potential new TB diagnostics.<br><br>**Perceived limitations**<br>Describe limitations to the generalizability of the analysis to inform health practice and policy decisions in other contexts or settings: South Africa has an almost unique health system in sub-Saharan Africa; and specific costs or 'real wold' performance of diagnostics is unlikely to similar elsewhere. Nevertheless, general lessons on the types of influences on resource use and outcomes may be useful to other policy makers, and those modelling Xpert MTB/RIF introduction elsewhere.<br><br>**Declarations of interest**<br>Pecuniary and non-pecuniary interests of the study contributors: None<br>Sources of funding and non-monetary sources of support for the conduct of the economic evaluation: Bill and Melinda Gates Foundation

**Principle 2: The comparator(s) against which costs and effects are measured should accurately reflect the decision problem**
**Minimum methodological requirement:** Comparative analysis for: a) the intervention and the base case comparator defined in the decision problem (Principle 1), which should be the intervention(s) currently offered to the population and b) the intervention and a "do nothing" comparator representing best supportive (non-interventional) care.<br><br>Describe intervention(s) currently offered to population:<br><br>Xpert MTB/RIF (Cepheid, Sunnyvale, CA, USA) is a rapid, molecular, cartridge-based test. In a laboratory based demonstration study done in three countries (Peru, Azerbaijan and South Africa), among drug-sensitive and multidrug-resistant pulmonary TB patients, a single Xpert MTB/RIF test on unprocessed sputum had an overall sensitivity of 92.2% (98.2% for smear positive and 72.5% for smear negative TB) and specificity of 99.2%. For rifampicin resistance the sensitivity was 97.6% and specificity was 98.1%. The sensitivity of Xpert MTB/RIF when used in routine microscopy centres for detection of smear positive and negative TB was similar and for RIF resistance was lower, although still good. The sensitivity of Xpert MTB/RIF test in diagnosing extrapulmonary TB is modest to good.<br><br>Given the high prevalence of HIV in South Africa, the NDOH took the decision to use Xpert MTB/RIF as the initial diagnostic test for all persons identified as potentially having TB. The diagnostic algorithm for TB is however complex, and should certain risk groups report a negative test result follow-on testing is required. As Xpert MTB/RIF also provides a signal for drug resistance the intervention includes the removal of the need for retreatment of TB in those who fail first line treatment – instead patients either receive first line treatment (if not resistant) or move on to further testing and treatment for multi-drug resistant TB (MDR-TB)<br><br>Describe chosen base-case comparator, including setting where comparator is administered:<br><br>The diagnosis of TB was previously conducted in South Africa using smear microscopy as the initial test (hereafter written as microscopy). Microscopy has a limited sensitivity, and cannot identify MDR-TB. Smear microscopy involves collecting sputum, staining and then identifying micro-bacteria under a microscope). Microscopy has a sensitivity of around 60% and a specificity of 98%. The sensitivity of microscopy is substantially lower when detecting TB in those living with HIV, given the lower levels of micro-bacteria observable.<br><br>Describe the availability of the comparator across the population being considered:<br><br>For those who have accessed health services and are identified as potentially having TB, smear microscopy is universally available in South Africa. However, South Africa does not identify many of those at risk of having TB. TB case detection is

estimated to be 69% of total TB cases in 2013. This is primarily thought to be due to a lack of recognition of TB symptoms and the low sensitivity of microscopy in those living with HIV.

Describe the "do nothing" comparator (i.e. minimum supportive care in the context of the decision problem):

The 'do nothing' comparator is not being examined in this case as there is sufficient evidence that microscopy (the 'standard of care' is cost-effective. Although not examined here, microscopy is likely to be a relatively cost effective intervention when compared to "do nothing" due to its low cost. However, if TB was not diagnosed then the likely 'do nothing' would be end of life care only.

Methods

Principle 3: An economic evaluation should consider all available evidence relevant to the decision problem.

Minimum methodological requirement: Apply a systematic and transparent approach to obtaining evidence and to judgements about evidence exclusion

Briefly describe approach used to obtain included evidence

Evidence on resource use, unit costs and mortality/ morbidity was collected from the pragmatic trial. Twenty laboratories were randomised to Xpert or microscopy arms. At two primary care clinics per laboratory, a systematic sample of adults giving sputum for tuberculosis investigation was enrolled. Vital status and proportion treated for tuberculosis were determined at six months. Participants starting tuberculosis treatment were identified by self-report and record review. Between June-November 2012, 4972 persons were screened, and 4656 (93.6%) enrolled and followed up for six months. Resource use (event) data was collected from a combination of participant interviews and case note extractions. Unit costs were collected using detailed micro-costing at study sites. Patient incurred costs were collected from a sub-sample of participants, in addition to other presumptive TB cases attending the clinics.

A very limited level of secondary data was required to estimate cost-effectiveness, as incremental costs and DALYs averted were estimated conservatively from within the trial period. The few areas were: HIV treatment costs and the life expectancies and disease weights used to estimate DALYs averted.

Please list documents included for review describing systematic review protocol or evidence search strategies:

There were no documents, but a systematic review (conducted and published by the same research team) was used to estimate HIV treatment costs

Briefly describe where evidence is incomplete or lacking in the following key areas:

a) Intervention and comparator performance/clinical effect:

We have mortality data for six months. We did not include potential longer term outcomes for those with MDR-TB given the lack of evidence in this area (see main paper).

b) Resource use and costs

We collected extensive data. Nevertheless (see below) it was not pragmatic to collect detailed data on patient/ household costs for all trial participants. We therefore had to impute data from our sample across the trial cohort.

c) Epidemiological data

As not a model based analysis there was less need for this. Nevertheless in estimating DALYs we have to use age-specific life expectancy. There is a lack of clarity on survival on ART in the South African setting. We make several approximations here for self-cure and median survival for those with different forms of the disease. We use standard assumptions, but there are few studies of the natural history of TB so the evidence base is weak in this area.

Minimum methodological requirement: Estimates of clinical effect of intervention and comparator(s) should be informed by systematic review of the literature

Describe methods for establishing clinical effect(s):

As above we sourced mortality data within the six month trial period, plus duration with TB symptoms as a proxy for morbidity. Any other parameters were taken from previous literature reviews used to estimate the cost-effectiveness of Xpert in a previous paper.

Minimum methodological requirement: Where a single study or trial forms the basis of the analyses, please describe steps taken to ensure relevant non-trial data has been incorporated into the analyses:

The aim of this study was to provide an empirical evidence of cost and effects in a 'real world' setting and compare to earlier modelled finding that were based on all available evidence. However, the purpose of this work was to present empirical findings for a specific population, and thus provide insightful evidence about the underlying dynamics/ mechanisms of costs and effects, rather than have generalizable results.

Is the stated decision problem specific to the particular context and time of the study or trial? **No**

**Principle 4: The measure of health outcome should be appropriate to the decision problem, should capture measurements of both length and quality of life, and should be generalisable across disease states.**
**Minimum methodological requirement**: Disability-Adjusted Life Years (DALYs) averted should be used.<br><br>Clearly describe the method of weighting used to inform DALYs:<br><br>We used the GBD 2010 weights for TB and TB/HIV co-infection<br><br>Discuss any important outcomes insufficiently captured by the DALY:<br><br>There are some issues re whether the TB/HIV are sufficient to cover those on ART and those not ART. We decided to take the full weight even if on ART.

**Principle 5: All differences between the intervention and the comparator in the expected resource use and costs of delivery to the target population(s) should be incorporated into the evaluation.**
**Minimum methodological requirement**: Estimates should reflect the resource use and unit costs/prices that may be expected if the intervention is rolled out to the population defined in the decision problem:<br><br>Please list quantities of resources separately from their unit costs/prices<br><br>This are described in the main paper, and complementary costing papers<br><br>Are capital and fixed costs annuitized over the period of implementation? Yes<br><br>If not, provide justification<br><br>Describe how the costs have been validated (e.g. corroboration with similar interventions in similar settings)<br><br>We did not look at other settings<br><br>Are costs converted to United States Dollars and local currency? Yes<br><br>If yes please state date and source of exchange rates: Will use the World Bank Database as a source
**Minimum methodological requirement**: Costs not incurred in study settings but likely if intervention is rolled out should be captured in the base case analysis<br><br>List all additional costs that may be incurred if the intervention were rolled out:<br><br>We have captured roll-out costs associated with Xpert scale-up at both the site and above site level. We estimated all the costs incurred above the laboratory level with the National Health Laboratory Service in South Africa, using their national data.<br><br>Were these costs included in the base case analysis? Yes<br><br>If not, provide justification:
**Minimum methodological requirement**: Cost all resource implications relevant to the decision problem, including estimations regarding donated inputs and out of pocket inputs from individuals<br><br>List all sources that will be used to fund the costs of the intervention(s) relevant to the decision problem (and list document(s) for review where these are fully costed):<br><br>As above in main paper and corresponding papers. We collected primary data on out of pocket costs.
**Minimum methodological requirement**: Analysis should include estimation of changes in costs estimates due to scalability<br><br>State any expected changes in costs due to scalability of the intervention (and list relevant document(s) for review):<br><br>We collected costs over 10 sites at different time points to estimate Xpert costs of over different service volumes; and estimated economies of scale.<br><br>We could not capture costs to scale up TB treatment, but have included the full cost as the incremental cost.

**Principle 6: The time horizon used in an economic evaluation should be adequate to capture all costs and effects relevant to the decision problem; an appropriate discount rate should be used to discount cost and effects to present values.**
**Minimum methodological requirement**: Lifetime time horizon should be used in the first instance. A shorter time horizon may be used where it can be shown that all relevant costs and effects are captured. Where the time horizon is > 30 years, the impact of lower discount rates should be explored in a sensitivity analysis.<br><br>State the time horizon over which costs and effects have been evaluated, including additional analyses if different time horizons have been explored:<br><br>The analysis captures all costs and effects within the trial period. We exclude longer term impact on those with MDR-TB due to lack of data. We are very careful in our inference concerning this issue.<br><br>If lifetime time horizon is not used, justify why and report impact of different time horizon(s):

The main bias this will create is two-fold. Firstly, if the intervention put people earlier on treatment or more people on treatment then we may see a differential different post six months. However, the trial did not find any significant difference in either of these two elements. The other main difference is MDR-TB, whereas Xpert may generate costs and save lives post the trial period differentially. The sample size of the trial (even at over 4000 enrolled presumptive TB cases) is not big enough to assess this issue. We have listed this as a study limitation.
**Minimum methodological requirement**: A 3% annual discount rate for costs and effects in the base case, with additional analyses exploring differing discount rates. Additional analysis should explore an annual discount rate that reflects the rate for government borrowings<br><br>State the discount rate used for costs and effects, and any additional analyses using different discount rates:<br><br>3% for costs and DALYS averted. Given time period have not done a sensitivity analysis around this, but have also estimated DALYs averted using 'no frills' DALYS with a 0% discount rate

Results

**Principle 7: Non-health effects and costs associated with gaining or providing access to health interventions that don't accrue to the health budget should be identified where relevant to the decision problem. All costs and effects should be disaggregated, either by sector of the economy or by whom they are incurred.**
**Minimum methodological requirement**: The base case analysis should reflect direct health costs and health outcomes.<br><br>Describe the incremental impact of the intervention on direct health costs and effects in the context of the decision problem (or list document(s) to review):<br><br>See main paper
**Minimum methodological requirement**: Additional analysis should adopt a disaggregated societal perspective to capture relevant non-health effects and costs that fall outside the health budget. The mechanism of inclusion will differ depending on the decision problem and context.<br><br>NA<br><br>Describe incremental impact of the intervention on non-health effects and costs that fall outside the health sector in the context of the decision problem:<br><br>NA<br><br>Explain and justify the mechanisms used to report impact of these cost and effects:<br><br>NA<br>If non-health effects and costs that fall outside the health sector are not included, please state reasons below and estimate the potential impact of these exclusions:<br><br>We also collected data on children leaving school – as a consequence of loss of work from TB (or seeking TB treatment), but did not incorporate or value this in the end analysis, due to the low numbers and issues with valuation.
**Minimum methodological requirement**: Where external funding or individual out-of-pocket (OOP) payments substitute for costs that would otherwise fall on a health budget, these costs should be included in the base case analysis, however the impact of excluding these should be explored in sensitivity analyses<br><br>State any alternative analyses exploring the impact of individual out-of-pocket payments and external funding (or refer to relevant document(s)):<br><br>We have not done this as we took a societal perspective

**Principle 8: The cost and effects of the intervention on sub-populations within the decision problem should be explored and the implications appropriately characterised.**
**Minimum methodological requirement**: Heterogeneity should be explored in population subgroups, where subgroup formation should be informed by: a) relevant effect of the intervention differs in different populations, b) characteristics of different populations that may influence the absolute health effects and c) characteristics that influence direct costs of provision or other associated costs across the constituency.<br><br>Describe subgroup characteristics and justify why particular groups were chosen for subgroup analysis:<br><br>We have not explored subgroups. Cost-effectiveness will be different for individual with HIV and individuals with MDR-TB. But in terms of policy implementation it would be infeasible (as many peoples HIV status is unknown) to implement Xpert MTB/RIF differentially in South Africa. While less relevant here, there is much controversy around explicitly addressing the differences in interventions for those with HIV and those without. For TB it is always going to be far less cost-effective to save the lives of those with HIV, as either the costs of ART are substantial, or life expectancy is short. This may of course be outweighed if the effect size difference is substantially favourable to those with HIV, but this issue raises ethical questions that then also need to be addressed possibly in some form of equity analysis. At present there is no clear guidance on how to do this.<br><br>In terms of comparing between control and intervention we have however adjusted our analysis for various covariates that were imbalanced including HIV status and Body Mass Index (BMI).

Minimum methodological requirement: Subgroup analysis should always be determined by: a) the evidence base regarding differences in relative effect, baseline risk or other characteristics and b) whether the differences are likely to have an important influence on costs and effects

Describe the evidence base used to determine subgroup specification:

We used the selection of covariates in the mortality analysis

Describe the cost effectiveness of the intervention in the different subgroups (or refer to relevant documents):

NA

Describe any subgroups with potentially important differences in costs and effects but that were excluded due to lack of evidence:

NA

Principle 9: The uncertainty associated with an economic evaluation should be appropriately characterised.

Minimum methodological requirement: The economic evaluation should explore uncertainty in the structure of the analysis

Describe how uncertainty in the structure of the analysis was explored (and refer to relevant documents):

We are used to conducting probabilistic sensitivity analyses in model based evaluations. Here we though we conducted standard statistic tests to establish whether there was any significant different in the intervention and control arm. The non-parametric TSB for cluster randomized trials (CRTs) was originally proposed by Davison and Hinkley was applied. This approach uses the conventional non-parametric bootstrapping but at the same time takes into account the clustering in CRTs. The TSB algorithm performs resampling in two stages. In the first stage it resamples clusters and in the second stage it resamples individuals within clusters. The resulting bootstrapped data sets can then be used to calculate the incremental cost-effectiveness ratio (ICER) and confidence intervals. We also generated acceptability curves using the outputs of this analysis. We also conducted a number of one way analyses on key areas like costs and discount rates.

Note any impact on final results.

Our results do find on average that Xpert MTB/RIF was cost-effective – but the result is highly uncertain, with the mean incremental cost and mean incremental DALYS averted being very close to zero. While the standard deviations of both cost and effect separate are reasonable, the combination of the two near zero means results many samples with negative ICERS. We do not therefore conclude that the intervention is cost-effective, but we describe the result as being cost neutral and impact neutral. While technically an intervention which has almost no impact on cost or effect may be cost-effective, for policy makers the message that no impact and no cost is perhaps more informative to their decision than the ICER.

Minimum methodological requirement: The economic evaluation should explore uncertainty due to source of parameters.

Describe how uncertainty due to source of parameters was explored (and refer to relevant documents)

As above – one way analysis on key areas of parameter uncertainty: ART costs and discount rates

Minimum methodological requirement: The economic evaluation should explore uncertainty due to precision of parameters**.** Uncertainty due to precision of parameters should be characterised using sensitivity analyses appropriate to the decision problem.

Describe how uncertainty due to precision of parameters was explored in sensitivity analyses (and refer to relevant documents):
As above

Principle 10: The impact of implementing the intervention on health budget and on other constraints should be clearly and separately identified.

Minimum methodological requirement: Budget impact analysis should estimate the implications of implementing the intervention on various budgets**.** Disaggregated and annualized budget impact analysis should be reported.

Describe budget implications for (and refer to any relevant documents):

At the initial stage of this project we used a budget analysis to estimate the costs of scaling up Xpert across South Africa. We primarily did this to support the NDOH, but also so we understood what may be driving uncertainty around costs, so we could ensure we collected the correct data. Given that the Xpert had been scaled up, and the trial was negative we did not repeat this at the end of the trial. The data from this study has been used to inform both the National Strategic Plan and the National TB Plan.

It should be noted that any access to expenditures or budgetary structures is very challenging in South Africa, so we were unable to analyse our budget impact against any affordability constraint

Describe significant non-budgetary constraints to the implementation of the intervention in the context of the decision problem

Our findings strongly suggest that there are many constraints that prevent a technology that should cost-effective if implemented having an impact. The proximal constraints are mainly care cascade issues, and links to complementary services (HIV care). The distal links are likely to be more systemic causes that prevent those suspected of having TB being followed through by staff, and may impact health seeking behaviour. It should be noted that mortality is extremely high for those who are co-infected, so relatively minor delays or lack of integrated service provision can be a major barrier to having a mortality impact.

Principle 11: An economic evaluation should explore the equity implications of implementing the intervention.
**Minimum methodological requirement**: There are various mechanisms available for assessing equity implications of an intervention. The method chosen should be appropriate to the decision problem and justifiable to the decision maker. Equity implications should be considered at all stages of the evaluation, including design, analysis and reporting.<br><br>Describe the method(s) used to incorporate equity implications (and refer to relevant documents):<br><br>We decided to conduct an equity analysis as this is a unique dataset of what happens to those with presumed TB in South Africa. As part of this post-hoc evaluation we wanted to examine who would be beneficiaries of this intervention; and whether it has had an anti-poverty impact as well as any impact on outcomes/ cost-effectiveness.<br><br>We also wished to inform methods on what may be feasible on a trial based dataset in this respect.<br><br>We used multiple imputation methods to impute patient/ household costs from our sub-sample to the whole database. We then estimate the numbers of individuals incurring catastrophic expenditures, poverty cases averted and changes to the poverty gap. We analysed these metrics disaggregated by social-economic status, age and sex; and compared between the control and intervention groups. This data is not presented in this paper, and is forthcoming.

Figure S1. A: The algorithm for the diagnosis of tuberculosis used by health care workers in facilities using Xpert (National Guidelines for TB diagnosis and management, 2014)[28]. B: The algorithm for the diagnosis of tuberculosis used by health care workers in facilities prior to capacitation. (South African National Guidelines for TB diagnosis and management, 2009)

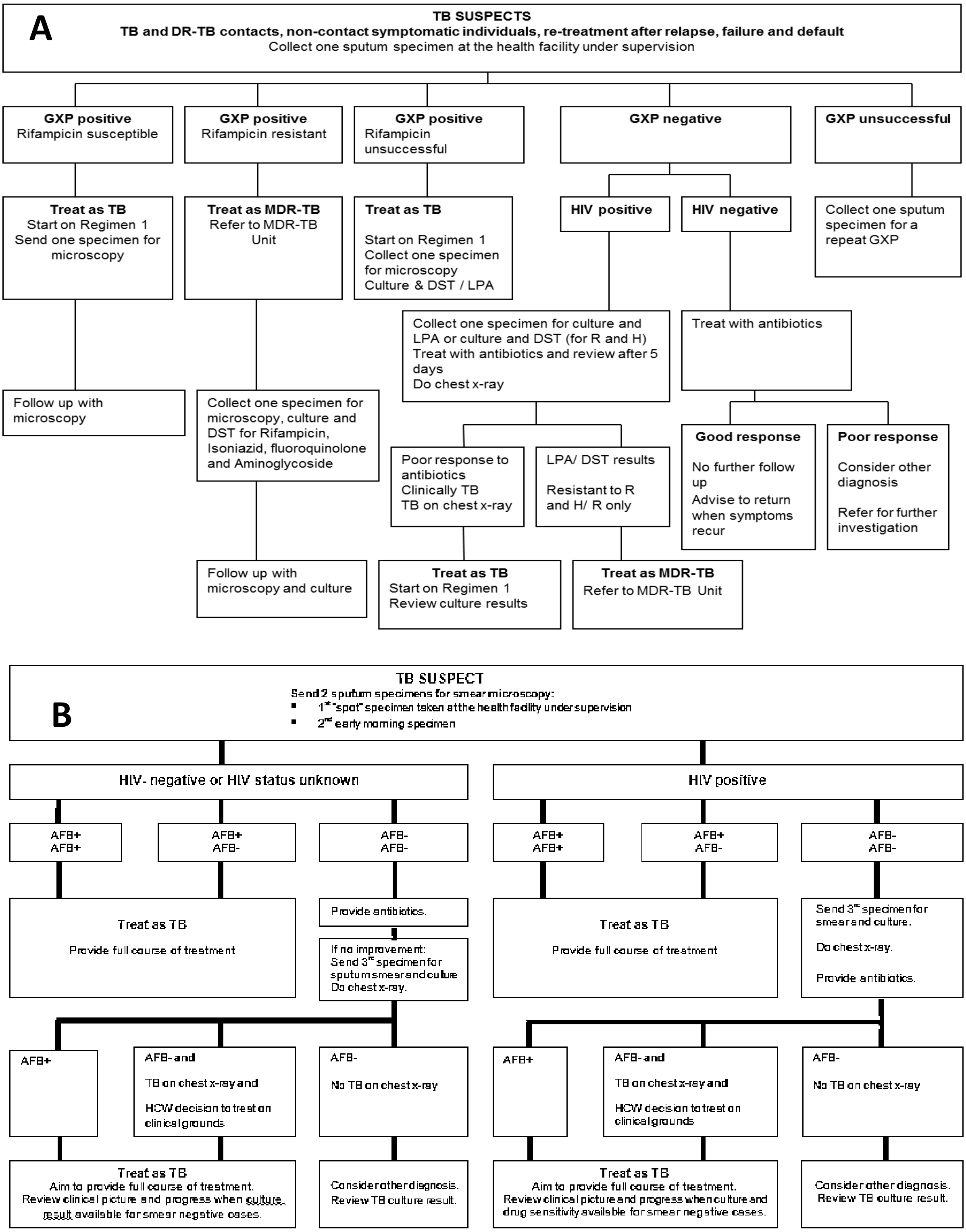

Summary of statistical methods used

We used two methods to address missing data; mean substitution and multiple imputation (Zhu 2014, Rubin 1987). [1,2] We used the mean substitution method to link mean 'per event' costs from our sub-sample of 351 to different health service use 'events' such as a health care visit or TB diagnostic test to create the simple imputation dataset. The mean substitution method imputes the missing values using the mean of the available observed values, coming from the sub-sample of 351.

We then applied multiple imputation to assign 'per event' costs from the same sub-sample to the total sample of 4656 XTEND participants by combining 10 imputed datasets. Multiple imputation was proposed by Rubin to analyse incomplete data under the assumption that data are missing at random. In our case, our data was missing by design, but our recovery of the data also assumes that the missing data in costs depend on completely observed variables in the dataset. The first step in the multiple imputation was performed using a linear regression framework based on key variables considered to be associated with the missingness mechanism and expected to predict costs. These included sex (binary), number of years at school (ordinal), self-reported HIV status (categorical), initial TB test result (binary), geographical location (binary: urban/rural), income (categorical), number of days with at least 1 symptoms (continuous), anyone else in household with a regular job (binary), household social economic status (SES) status (categorical – derived through principal component analysis), number of health care visits (continuous), country of birth (categorical), age (continuous), province (categorical) and distance from residence to clinic (ordinal). We then collapsed the imputed datasets to generate the multiple imputation dataset. Given the high number of zero observations in our sub-sample for some 'per event' costs, we applied a two-part model, which first predicts non-zero costs for each type of patient event, and then predicts the level of costs for events with a non-zero cost.

The cost and cost-effectiveness analyses reflected the clustered design and baseline imbalance between study arms.[3] For the cost analysis, we used a two-stage cluster level analysis with bootstrapping. We applied a multiple ordinary least square (OLS) regression model with robust standard errors using 200 bootstrap replications and adjusting for HIV status, socio-economic status (SES), ethnicity, education, marital status, age group, sex, province, body mass index (BMI) group, and number of symptoms. We calculated the predicted cost for each patient and subsequently collapsed the data across cluster and calculated the residuals (difference of observed and predicted) costs for each cluster. In the second stage, we ran an ordinary least squares (OLS) regression analysis at the cluster level to assess differences in costs between Xpert and microscopy arms using the residuals as the dependent variable. We used this method to estimate incremental costs between the two study arms.

For our cost-effectiveness analysis, we used a modified version of the traditional non-parametric two-stage bootstrapping (TSB) proposed by Davison and Hinkley[4] to estimate cost-effectiveness ratios per death and DALYs averted, using 10,000 replications. The traditional TSB algorithm performs resampling with replacement in two stages. In the first stage, it resamples clusters and in the second stage it resamples individuals within clusters. A 'shrinkage estimator' is used to correct for potential overestimation of variance. Bootstrap datasets are then constructed combining the resampled shrunken cluster means with resampled individual-level residuals. In the modified version, proposed by Gomes et al (2012), each cluster mean is combined with individual residuals drawn from the same cluster, after the 'shrinkage' correction is applied, in order to preserve the relationship between the cluster mean and the covariate information within the cluster in the bootstrap datasets. The following steps describe the modified TSB algorithm used, using 10,000 replications[3]:

1. For i in 1 to n_j (individuals in cluster j).
2. For j in 1 to M_k (clusters in treatment k).
3. For k in 1 to 2 (treatments).
4. Calculate shrunken cluster means, ($\hat{x}_j c$ and $\hat{x}_j e$), for costs and outcomes
5. Calculate standardised individual-level residuals, ($\hat{z}_{\text{cost},ji}$ and $\hat{z}_{\text{outcome},ji}$), for costs and outcomes.
6. Randomly sample (with replacement) M_k pairs of cluster means, ($x^*_{\text{cost},j'}$ and $x^*_{\text{outcome},j'}$), from the shrunken cluster means calculated in step 4
7. Within each resampled cluster, randomly sample (with replacement) $\sum_{j'=1}^{M_k} n_{j'}$ pairs of residuals, ($z^*_{\text{cost},i'}$ and $z^*_{\text{outcome},i'}$), where i'=1, . . . $\sum_{j'=1}^{M_k} n_{j'}$, from the standardised residuals calculated in step 5.

8. Re-construct the sample, $(y^*_{\text{cost},j'i'}, y^*_{\text{outcome},j'i'})$, by adding the shrunken cluster means from step 6 and the standardised residuals from step 7. For example, for costs $y^*_{\text{cost},j'i'} = x^*_{\text{cost},j'} + z^*_{\text{cost},i'}$ where i'=1… $n_{j'}$, and likewise for outcomes, and this can be called a 'synthetic' sample.
9. Incorporate the covariate $w_{j'i'}$ into each synthetic sample as follows: $(y^*_{\text{cost},j'i'} + w_{j'i'}$, $y^*_{\text{outcome},j'i'} + w_{j'i'})$. Note that the set of covariates can differ for cost and outcomes. Adjusted costs (as described above) and adjusted deaths using the adjusted analysis on outcomes as outlined in the main trial paper – see Table S3).
10. Repeat steps 4 to 9 for each treatment arm and stack these 'synthetic' samples into a single bootstrap sample.
11. Replicate steps 6 to 10 *R* times to construct *R* bootstrap samples.

References

1. Zhu X. Comparison of four methods for handing missing data in longitudinal data analysis through a simulation study. *Open Journal of Statistics* 2014; **4**: 933-944.
2. Rubin DB. Multiple imputation for nonresponse in surveys. New York: *John Wiley & Sons Inc,* 1987.
3. Gomes M, Grieve R, Nixon R, Ng ES, Carpenter J, Thompson SG. Methods for covariate adjustment in cost-effectiveness analysis that use cluster randomised trials. *Health Econ* 2012; **21**: 1101–18.
4. Davison AC, Hinkley DV.. Bootstrap Methods and Their Application. Cambridge, UK: *Cambridge University Press*, 1997.
